# Markov clustering versus affinity propagation for the partitioning of protein interaction graphs

**DOI:** 10.1186/1471-2105-10-99

**Published:** 2009-03-30

**Authors:** James Vlasblom, Shoshana J Wodak

**Affiliations:** 1 Molecular Structure and Function Program, Hospital for Sick Children, 555 University Avenue, Toronto Ontario, M5G 1X8, Canada; 2 Department of Biochemistry University of Toronto, 1 King's College Circle, Toronto Ontario, M5S 1A8, Canada; 3 Department of Molecular Genetics, University of Toronto, 1 King's College Circle, Toronto Ontario, M5S 1A8, Canada

## Abstract

**Background:**

Genome scale data on protein interactions are generally represented as large networks, or graphs, where hundreds or thousands of proteins are linked to one another. Since proteins tend to function in groups, or complexes, an important goal has been to reliably identify protein complexes from these graphs. This task is commonly executed using clustering procedures, which aim at detecting densely connected regions within the interaction graphs. There exists a wealth of clustering algorithms, some of which have been applied to this problem. One of the most successful clustering procedures in this context has been the Markov Cluster algorithm (MCL), which was recently shown to outperform a number of other procedures, some of which were specifically designed for partitioning protein interactions graphs. A novel promising clustering procedure termed Affinity Propagation (AP) was recently shown to be particularly effective, and much faster than other methods for a variety of problems, but has not yet been applied to partition protein interaction graphs.

**Results:**

In this work we compare the performance of the Affinity Propagation (AP) and Markov Clustering (MCL) procedures. To this end we derive an unweighted network of protein-protein interactions from a set of 408 protein complexes from *S. cervisiae *hand curated in-house, and evaluate the performance of the two clustering algorithms in recalling the annotated complexes. In doing so the parameter space of each algorithm is sampled in order to select optimal values for these parameters, and the robustness of the algorithms is assessed by quantifying the level of complex recall as interactions are randomly added or removed to the network to simulate noise. To evaluate the performance on a weighted protein interaction graph, we also apply the two algorithms to the consolidated protein interaction network of *S. cerevisiae*, derived from genome scale purification experiments and to versions of this network in which varying proportions of the links have been randomly shuffled.

**Conclusion:**

Our analysis shows that the MCL procedure is significantly more tolerant to noise and behaves more robustly than the AP algorithm. The advantage of MCL over AP is dramatic for unweighted protein interaction graphs, as AP displays severe convergence problems on the majority of the unweighted graph versions that we tested, whereas MCL continues to identify meaningful clusters, albeit fewer of them, as the level of noise in the graph increases. MCL thus remains the method of choice for identifying protein complexes from binary interaction networks.

## Background

Protein-protein interactions play a key role in cellular processes and significant efforts are being devoted world wide to characterizing such interactions on the scale of whole genomes (for review see [1,2]). Genome scale data on protein interactions are typically obtained using experimental methods for detecting binary interactions[3,4], or by affinity purifications of tagged proteins coupled to analytical methods for identifying the co-purified partners [5-7]. These data are in general represented as large networks, or graphs where hundreds or thousands of proteins are linked to one another [8-10]. For a recent review of network analysis techniques as applied to protein interaction networks, see [11].

It is well known however that proteins tend to function in groups, or complexes, which in the yeast *S. cerevisiae *contain on average 4.7 different types of subunits [12,13]. An important goal has therefore been to reliably identify protein complexes from the protein interaction graphs. This task is commonly carried out using graph clustering procedures, which aim at detecting densely connected regions within the interaction graphs.

Clustering is an unsupervised learning method that tackles the task of producing an intrinsic grouping of data elements on the basis of some metric (a 'distance' or similarity measure between elements). It requires solving an optimization problem, which is usually achieved with the help of heuristic algorithms whose ability to approximate the best solution (global minimum) may vary widely[14]. Their application in the context of protein interaction networks encounters the additional problem of dealing with the significant level of background noise in these networks[15] (e.g. spurious interactions that have no biological meaning). Dealing with a high level of noise is a major challenge for clustering procedures, as this requires mitigating the effect of noise by various means – for example by taking into account the topology properties of the network, either during the clustering process or by modifying the distance metric to incorporate such properties prior to clustering.

There exists a wealth of clustering algorithms of which hierarchical clustering (for review see [16,17]) and K-means[18,19] are classical examples. More recently a variety of other algorithms have been proposed[20], and some of these have been applied to the identification of highly connected nodes in protein interaction graphs[7,21].

So far, one of the most successful clustering procedures used in deriving complexes from protein interaction networks seems to be the Markov Cluster algorithm (MCL)[22]. Unlike most hierarchical clustering procedures, this algorithm considers the connectivity properties of the underlying network. It has been used to derive complexes from protein interaction data in two recent comprehensive analyses of the yeast interactome [7,21]. Furthermore, in a recent benchmark carried out by Brohée et al[15], MCL was shown to be especially effective for clustering protein interactions in that it possesses a high degree of noise-tolerance in comparison to other algorithms such as the Molecular Complex Detection (MCODE)[23] and Super Paramagnetic Clustering (SPC)[24].

Over a year ago, a novel promising clustering procedure termed Affinity Propagation (AP) was proposed [25]. Affinity propagation identifies representative examples (exemplars) within the dataset by exchanging real-valued messages between all data points. Points are then grouped with their most representative exemplar to give the final set of clusters. AP was applied to a variety of problems including face recognition, and gene identification from putative exons using microarray data, and was shown to be faster and more accurate than the K-Centers[18] clustering algorithm. A subsequent note suggested however, that AP was similar to the earlier vertex substitution heuristic (VSH), and that it did not perform any better[26]. This prompted the AP authors to provide evidence that AP outperforms VSH on large problems – where it runs much faster, and was more accurate than several clustering algorithms tested[27].

In view of the interest in applying efficient clustering procedures to biological networks in order to identify and characterize functional modules, this paper expands the analysis of Brohée et al[15] to the comparison of the AP and MCL algorithms. Such comparison has not been previously reported.

Following Brohée et al, we first derive an unweighted network of protein-protein interactions from a set of up-to-date hand curated protein complexes from *S. cervisiae*[28] and evaluate the performance of the two clustering algorithms in recalling the annotated complexes. In doing so the parameter space of each algorithm is sampled in order to select optimal values for these parameters, and the robustness of the algorithms is assessed by quantifying the level of complex recall as interactions are randomly added or removed to the network to simulate noise.

To test performance on a more realistic weighted protein interaction graph, we also apply the two algorithms to the high confidence consolidated protein interaction network of *S. cerevisiae *recently derived by Collins et al[29], and to versions of this network in which varying proportions of the links have been randomly shuffled. The computed clusters are compared to the same set of curated *S. cerevisiae *complexes in order to assess the robustness of the two algorithms.

The comparative analysis on the unweighted networks proposed here has the advantage of representing a self-consistent approach, in which information on a predefined number of cliques is used to build the network, and hence the expected result from partitioning this network is well defined. The choice of the weighted high confidence consolidated network of *S. cerevisiae *recently derived from purification data also enables to quantify the performance of the clustering procedures by comparing computed clusters to the annotated complexes. Such quantification is difficult with *S. cerevisiae *protein interaction networks built using yeast two hybrid data, because these interactions differ significantly from co-complex interactions[30]. Partitioning this network using any method is hence unlikely to yield clusters comparable to complexes. The much larger human protein interaction networks compiled from different sources and stored in databases such as HPRD (~50,000 interactions), would not serve our purpose either, given the still limited number of fully annotated human protein complexes against which the clustering results can be compared.

### The clustering algorithms

The Markov clustering algorithm (MCL) simulates random walks on the underlying interaction network, by alternating two operations: expansion, and inflation. First, loops are added to the input graph – by default, the loop weight for each node is assigned as the maximum weight of all edges connected to the node – and this graph is then translated into a stochastic "Markov" matrix. This matrix represents the transition probabilities between all pairs of nodes, and the probability of a random walk of length n between any two nodes can be calculated by raising this matrix to the exponent n – a process referred to as expansion. As higher length paths are more common between nodes in the same cluster than nodes within different clusters, the probabilities between nodes in the same complex will typically be higher in expanded matrices. MCL further exaggerates this effect by taking entry wise exponents of the expanded matrix, and then rescaling each column so that it remains stochastic – a process called inflation. Clusters are identified by alternating expansion and inflation until the graph is partitioned into subsets so that there are no longer paths between these subsets.

Affinity Propagation (AP) identifies cluster centers, or exemplars, from the graph, which in some sense are a representative member of the cluster. Initially, all nodes are considered as exemplars, though each node is manually assigned a "preference" that it should be chosen as an exemplar. If no prior knowledge is available on which nodes should be favored as exemplars, then all nodes can be assigned the same preference value – where the magnitude can be used to control cluster granularity. For each node i and each candidate exemplar k, AP computes the "responsibility" r(i, k), which indicates how well suited k is as an exemplar for i, and the "availability" a(i, k) reflecting the evidence that i should choose k as an exemplar.



Where the matrix s(i, k) denotes the similarity (eg. edge weight) between the two nodes i and k, and the diagonal of this matrix contains the preferences for each node. The above two equations are iterated until a good set of exemplars emerges. Each node i can then be assigned to the exemplar k which maximizes the sum a(i, k) + r(i, k), and if i = k, then i is an exemplar. A damping factor between 0 and 1 is used to control for numerical oscillations.

## Results and discussion

### Performance on unweighted protein interaction graphs

**Figure 1:**
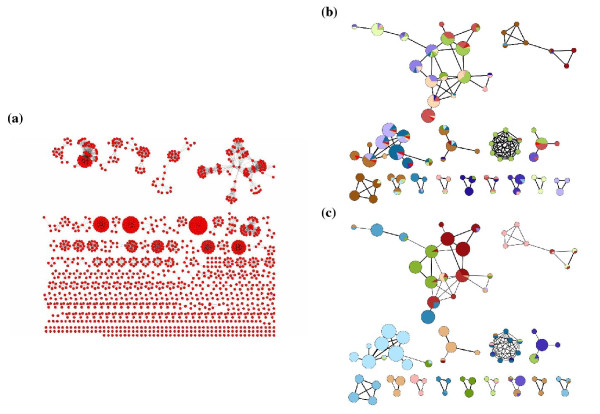
**Original unweighted protein interaction graph and graphs of curated complexes linked through their shared components**. (a)Unweighted protein interaction graph comprising 1628 proteins (nodes) and 11 249 interactions (edges) generated from the 408 hand curated complexes of *S. cerevisiae*[28]. (b, c)two copies of a portion of the graph in (a), where complexes (nodes) are linked to one another whenever they share at least 2 components, and the node size is proportional to the number of unique proteins each complex contains. (b)and (c)have the AP and MCL clusters respectively mapped onto the curated complexes, so that pie charts show proportions of complex components that are annotated to the same AP or MCL cluster. The mapped clusters are computed from versions of the original unweighted network shown in (a) in which 20% of the edges were randomly added and 20% randomly removed. Complexes whose components distribute among many clusters appear as multi-colored pie graphs, whereas those that are annotated to the same cluster appear solid-colored. The bright red color indicates the proportion of components that were assigned to singleton clusters by the AP or MCL algorithm. All the comparisons were performed with partitions obtained by optimizing the MCL and AP parameters respectively (see Methods). The pie graphs were generated using the GenePro plugin[32] for Cytoscape[31].

Both algorithms are first applied to partition unweighted protein interaction graphs. The original version of these graphs was built from a set of 408 *S. cerevisiae *protein complexes hand curated in-house[28] (see Additional File 1). In this graph, nodes represent individual proteins from these complexes, and any two proteins belonging to the same complex are linked by an edge. Figure 1a illustrates this graph as rendered by the Cytoscape[31] network visualization and analysis software. This rather disjoint graph is comprised of 11,249 interactions and 1,628 proteins, where the majority of the proteins are linked only to members of the same complex, forming distinct cliques, and only a small fraction are linked to members of different complexes. This graph is clearly a less challenging test for clustering procedures than protein interaction networks built from experimental data, since those networks include an appreciable level of spurious links (False Positive links). Networks built from experimental data typically feature more links between proteins in different complexes and not all members of a given complex are always linked to one another. To better mimic these more realistic networks we randomly add or remove links to this original network in various proportions, as done by Brohée et al. [15] thereby generating different versions of the original network which include varying levels of noise, representing different proportions of False Positives (FP) and False Negatives (FN) (links deleted from the graph, but present in the original network).

The MCL and AP clustering procedures were each applied to the different versions of the networks and the correspondence between the computed clusters and the original 408 curated complexes was evaluated for each network version. The correspondence was quantified using the Geometric Accuracy (*Acc*) and Geometric Separation (*Sep*) criteria as previously defined[15]. *Acc *is computed as the geometric mean of the Positive Predictive Value and Sensitivity with which the clusters recall the original complexes. The *Sep *parameter is defined as the geometric mean of two quantities that measure how cluster components are on average distributed amongst complexes and how complex components are distributed among clusters, respectively (see Methods for further details).

To enable as fair a comparison as possible, values of the adjustable parameters in each clustering algorithm were selected so as to maximize the sum of the *Acc *and *Sep *values for the clusters computed from each network (see Methods).

**Figure 2:**
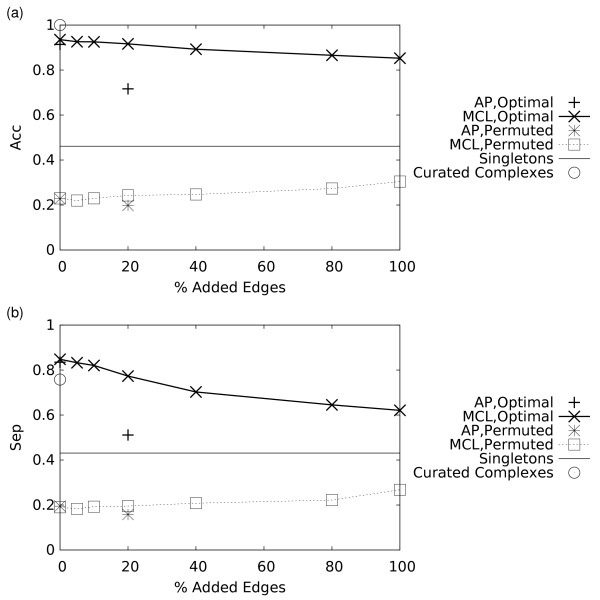
**Effect of random addition of edges to the original unweighted protein interaction graph on the performance of the MCL and AP algorithms**. Performance is evaluated by plotting two parameters, the Geometric Accuracy (*Acc*) (a), and Separation (*Sep*) (b). The random addition of varying proportions of edges mimics noise created due to varying proportions of False Positive interactions (spurious interactions). For AP, only those points where the algorithm converged are plotted. Definitions of *Acc *and *Sep *are given in Methods. The open circle marks the *Acc *and *Sep *values achieved by the curated complexes used to generate the original protein interaction graph, as measured against themselves – note that separation is < 1 due to shared components between complexes. Dashed lines indicate the values obtained from random graphs used as controls (see Methods). The solid horizontal line shows the *Acc *(a) or *Sep *(b) values achieved by not grouping any proteins (i.e. a "clustering" that consists entirely of singletons).

**Figure 3:**
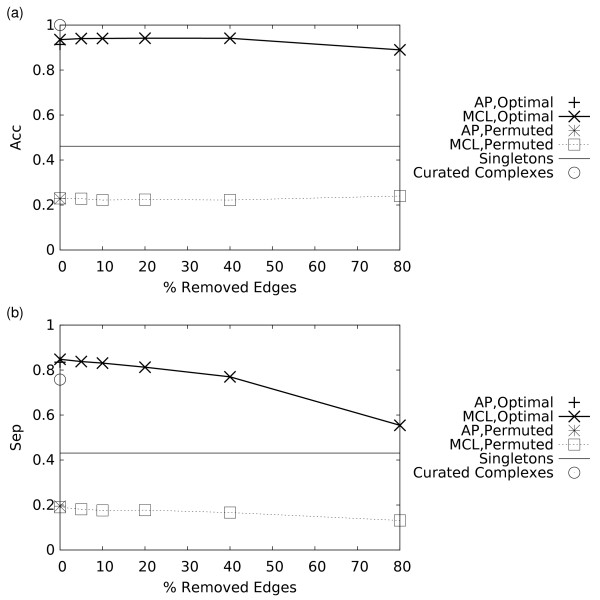
**Effect of random removal of edges from the original unweighted protein interaction graph on the performance of the MCL and AP algorithms**. Performance is evaluated by plotting two parameters, the Geometric Accuracy (*Acc*) (a), and Separation (*Sep*) (b). The solid horizontal line, dashed lines, and the open circle are as described in the figure 2 caption.

Figures 1b and 1c present a visual overview of the results obtained from an unweighted network, derived from the original network by adding 20% of the edges, and rendered using the GenePro[32] plugin for Cytoscape. They show that the AP clusters are more fragmented than those obtained with MCL, as components annotated to the same curated complex are often distributed among several AP clusters, whereas the MCL clusters tend to map more fully into the curated complexes. This result is summarized by the *Acc *and *Sep *parameters listed in the Additional File 2. To further understand how each algorithm handles noise, simulated here by random addition and subtraction of graph edges, we focus on the effects of either adding (Figure 2) or removing (Figure 3) edges. While AP and MCL yield solutions with virtually identical Acc and Sep values for the original network (zero noise level), the AP algorithm did not converge for most of the noisier networks. The one with 20% random edge addition was among the few for which it did converge, but the *Acc *and *Sep *values of the resulting clusters were much lower than those obtained with MCL on the same network. AP also did not converge for the majority of networks with simultaneous random edge addition and removal (Additional File 2). In contrast, MCL generated clustering solutions with relatively high *Acc *and *Sep *at all noise levels. Interestingly however, for networks containing high noise levels, the MCL clusters group only a fraction of the proteins comprising the interaction network, leaving the remaining proteins ungrouped (singletons) (Additional File 2).

We also tested AP on an unweighted network of 15 982 human protein-protein interactions comprising 5850 unique proteins, annotated as experimentally characterized using affinity capture or reconstituted complexes in version 2.0.50 of the BioGRiD database[33]. Similar to the results obtained for unweighted networks to which artificial noise was added, AP did not converge for this more realistic network derived from inherently noisy experimental data. MCL produced clusterings containing between 663 and 1566 clusters, depending on the inflation value. A detailed analysis of these clusters is outside the scope of this report, but the size distributions of the clusters in the MCL partitions produced at various inflation values (Additional File 3) indicate that they are not all trivial singleton or extremely large clusters.

The *Acc *and *Sep *were also evaluated for the 408 curated complexes directly. As expected,* Acc*, which quantifies the maximum extent of overlap between complexes and clusters – and vice versa – is 1 for these complexes (Figures 2a, 3a). Lower *Acc *values are obtained for the partitions derived by both clustering algorithms – largely due to shared components in the original complexes, which can obscure their detection, especially for smaller clusters. In contrast, shared components lower the *Sep *values of the original complexes, and hence as the clustering algorithms partition the graphs they can achieve higher *Sep *values at low noise levels (Figures 2b, 3b).

These results depart sharply from those expected for random partitions, as also illustrated in Figures 2a, b, 3a, and 3b. Random partitions were generated by randomly permuting the assignments of the proteins to clusters within the MCL and AP predictions.

### Performance on a weighted biological protein interaction graph

A second series of tests was performed using interaction graphs built from the consolidated network of Collins et al[29], where each protein-protein link has an associated confidence score ranging in values from 0 to 1. As in previous studies[21,29], only the high confidence portion of the network was considered, comprising links whose scores are above a confidence threshold of 0.38[21]. The resulting network comprised 12,035 interactions and 1,921 proteins. Since this network represents predicted associations from data derived in two recent high-throughput experimental studies[6,7], some noise will naturally be present. We did however generate noisier versions of this network by randomly shuffling increasing fractions of edges, and re-evaluating the results for each of these versions. As for the performance tests on the unweighted graphs, the parameters of each algorithm are adjusted so as to optimize the correspondence with the curated complexes, by maximizing the sum of the *Acc *and *Sep *values as done above for the comparative analysis on the unweighted graphs.

**Figure 4:**
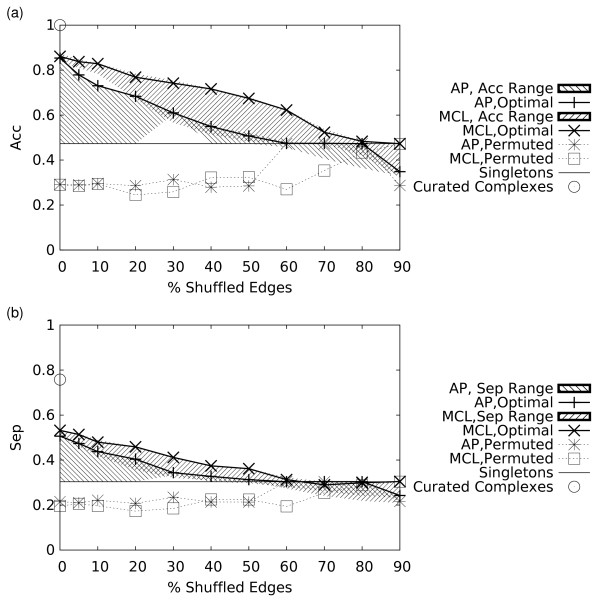
**(a) Geometric accuracy (*Acc*) and (b) Separation (*Sep*) as varying numbers of edges are shuffled within the weighted network**. Shaded regions indicate the range of *Acc *(a) or *Sep *(b) values achieved by each clustering algorithm as their parameter values were varied. The open circle marks the *Acc *and *Sep *values achieved by the curated complexes used to generate the unweighted networks. The solid horizontal line and dashed lines are as described in the caption of Figure 2.

On this more realistic network, both AP and MCL were able to predict clusters at all the tested noise levels. The results illustrated in Figure 4, show that, as expected, the *Acc *value tends to decrease with added noise for both algorithms, and that the *Acc *of MCL is higher than AP at all noise levels. The shaded areas in Figure 4 indicate the ranges in the *Acc *and *Sep *values covered by solutions obtained by varying the parameters of the AP and MCL algorithms, respectively. It can be seen from this figure that our parameter selection procedure was successful in identifying parameter values that approximately maximize the *Acc *and *Sep *measures independently at all noise levels.

**Figure 5:**
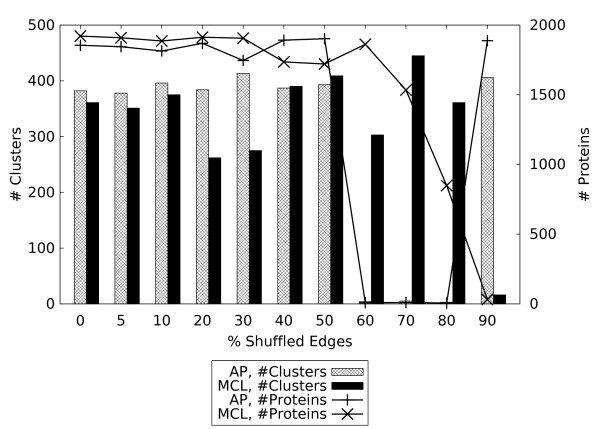
**Number of clusters and proteins included in these clusters obtained by each algorithm on the weighted network at different noise levels, after removing singletons**. At higher noise levels, the optimal AP solution in terms of *Acc*+*Sep *consists almost entirely of singletons, though coarser solutions also exist (see text, and Additional File 5).

We also see that at high levels of noise, the results are no longer meaningful as the clusters predicted by either algorithm consist almost entirely of singletons. Both algorithms have a slowly decreasing *Sep *as progressively more edges are shuffled. When no artificial noise is introduced, both algorithms are roughly comparable in terms of *Acc*, although the AP solution has slightly lower *Sep *and incorporates 61 fewer proteins than MCL (see Figure 5), which are classified as singletons. Examples of complexes recovered by MCL, but not AP are given in Figure S3 of the Additional File 4. As the level of artificial noise increases to 10%, both algorithms maintain approximately the same number of clusters and proteins. At 20%–30% noise, the optimal MCL solution in terms of *Acc+Sep *happens to correspond to a much coarser clustering than that obtained with AP (smaller number of clusters in Figure 5). However, using different Inflation values can generate solutions featuring finer granularities with only a minor decrease in *Acc+Sep *(Additional File 5). Overall, at around 60–70% noise predictions from both algorithms begin degenerating into singletons. The relative performance of MCL and AP does not depend on the objective function *Acc+Sep*. We verified indeed that at any preference value used for AP, clustering solutions produced by MCL have higher *Acc *and equivalent or higher *Sep *values at all inflation values tested (Figure S1 in Additional File 4).

**Figure 6:**
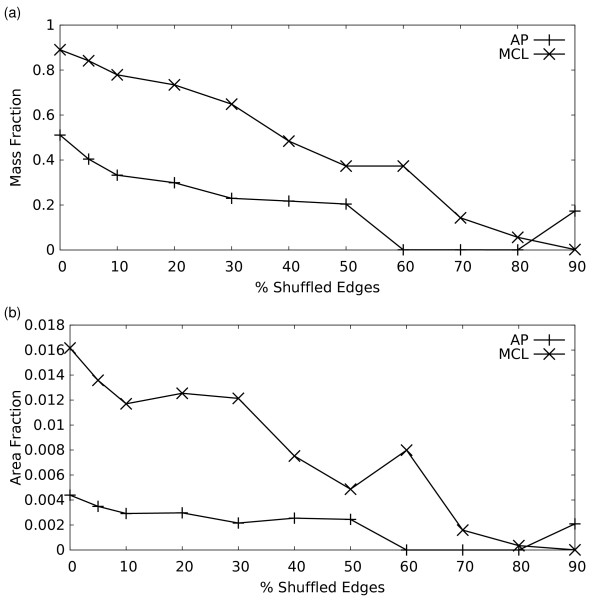
**Mass and Area Fractions of the AP and MCL solutions at varying noise levels**. These values assess the intrinsic efficiency of a clustering in terms of the amount of edge mass captured, with higher values indicating improved efficiency (see Methods for detail). The Mass fraction summarizes how much of the total edge weight is captured within clusters. The Area fraction is a measure of cluster granularity, such that higher area fractions correspond to coarser clusterings (See text).

To gain insight into how the MCL and AP clustering solutions change as edges are randomly shuffled, we plotted the mass fraction and area fraction (Figures 6a, b) for the optimal clustering at each noise level as found above. The mass fraction of a clustering solution for a weighted graph is simply the fraction of the total edge weight that is entirely contained within clusters. The area fraction assumes that each identified cluster is a clique, and measures the number of these clique edges relative to the total number of edges in a clique of all nodes (see Methods). We see that for both algorithms, the mass fraction decreased as edges are shuffled – which is expected given that formerly intra-complex edges are being reassigned as inter-complex edges during the shuffling. The area fractions also decreased for both algorithms, suggesting more granular clusterings.

**Figure 7:**
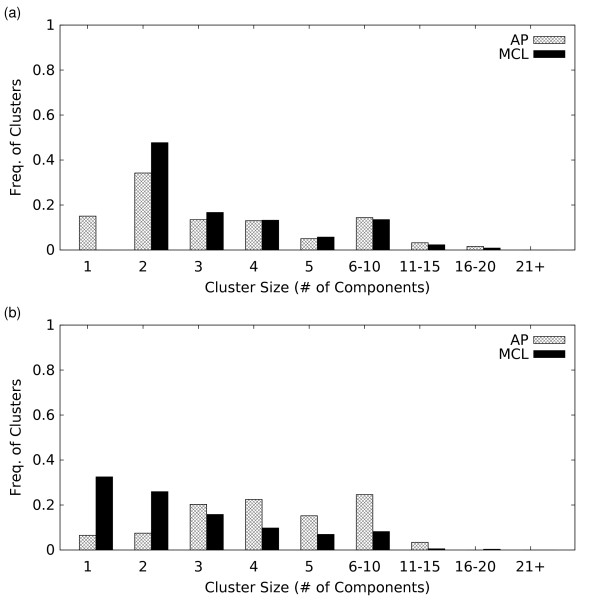
**Size distributions of clusters produced by the AP and MCL algorithm using the weighted network, when a) no edges are shuffled and b) when 40% of edges are shuffled**.

Overall we find that MCL tends to generate a more granular clustering in the presence of noise (Figures 7a, b) – although at very low noise levels AP produces more singletons and 2-members clusters than MCL. We also find that the higher *Acc *obtained with MCL in the presence of noise is maintained across the entire range of complex sizes (Figure S2b in Additional File 4), so that MCL's ability to recapitulate the curated complexes even at high noise levels (40% artificial noise) is better than AP for complexes of all sizes. In contrast, AP generally produces coarser clusterings as noise is increased, although the number of very large (16 or more components) clusters does decrease, reducing the overall area fraction.

These results, together with the superior *Acc *and *Sep *values obtained with MCL at high noise levels suggest that this algorithm is a better choice for weighted protein interaction networks.

## Conclusion

In summary, our analysis has shown that the MCL procedure is significantly more tolerant to noise and behaves more robustly than the AP algorithm. The advantage of MCL over AP is dramatic for unweighted protein interaction graphs, as AP displays severe convergence problems on the majority of the unweighted graph versions that we tested, whereas MCL continues to identify meaningful clusters, albeit fewer of them, as the level of noise in the graph increases. It is possible that AP as it stands, is not suitable for unweighted networks (as discussed below), although this is not specified in the instructions for using the program or in the original publication[25].

On weighted graphs constructed using data from high throughput experiments believed to be incomplete and usually quite noisy, the difference in performance is also notable. MCL achieves higher *Acc *and equivalent or better *Sep *at all significant noise levels. Furthermore, at low to moderate noise levels, these solutions include more proteins than AP. Parameters for either algorithm can be adjusted to affect the final granularity of the cluster, but either the *Acc *or the *Sep *will be lower.

Thus for physical interaction networks, we find that MCL outperforms AP in terms of its ability to generate meaningful partitions. The other cited advantages of the AP algorithm, namely its speed and ability to tackle very large networks, play only a minor role in the present application. Indeed both MCL and AP run very fast (< 10 seconds) on the weighted consolidated network of 12,035 interactions and 1,921 proteins. As noise is added to this network, AP can also fail converge at certain preference values (Figure S1 in Additional File 4 and Additional File 5), and it can be difficult to determine which parameters will lead to convergence. For example, AP didn't converge at any of the Preference values tested for unweighted networks with edges randomly removed. On weighted networks with 30% noise, the algorithm converged at Preference values 0.65 and 0.9 only (Additional File 4). Thus for this application, one difficulty in using AP is to determine an appropriate interval and level of granularity for searching Preference values. The AP authors provide tools to assist in choosing sensible Preference intervals, but not for choosing granularity. In situations where AP does not converge, the authors recommend increasing the Damping factor, the maximum number of iterations, and the number of iterations required for convergence – although increasing these parameters can increase the runtime of the algorithm.

The MCL algorithm effectively considers both edge weight and graph topology (connectivity) information. AP, on the other hand, can fail in situations where high weight edges connect two clusters. Consider the artificial situation where two cliques, A and B, are connected by a single, relatively high weight edge. If one of the nodes comprising this edge is an exemplar in clique A, the adjacent node in clique B may be incorrectly assigned to A by AP, despite being highly connected to members of B. This suggests that MCL achieves its robust performance by always considering network topology, whereas AP relies in part on the 'distance metric' (edge weight) to capture this information. To overcome this limitation one could define a modified distance metric that simultaneously captures both the propensity of two proteins to interact and the graph topology, and re-run AP on the modified graph. To some extent, the PE score is such a metric as higher scores are assigned to proteins that repeatedly co-purify together in affinity capture experiments, and lower scores are assigned to non-specific interactions that occur between promiscuous proteins. Indeed, on the PE weighted network of Collins et al, the performance of AP is much closer to that of MCL when the network is unperturbed, as randomly shuffling edges distorts the topology information contained in the edge weights. In the unweighted network, where no topological information is captured by the distance metric, AP is only able to successfully cluster unperturbed networks with very few inter-complex edges (shared components).

As noted in [27], the relative accuracy and performance of clustering algorithms can vary greatly for different datasets, and this report makes no attempt to address the breadth of problems for which one algorithm outperforms the other.

## Methods

### Building the protein interaction graphs

The unweighted interaction graph was defined by considering all possible pairs of proteins that were annotated to the same complex within a gold-standard set of yeast protein complexes[28] (Additional File 1). Each edge was assigned a weight of 1. The resulting network comprised 11,238 interactions (edges) and 1624 proteins (nodes). For AP, the input pairwise 'similarities' were defined twice for every pair of proteins i, j as S(i, j) = S(j, i) = 1 if protein i and j were annotated to the same gold standard complex.

The weighted interaction network is that derived by Collins et al[29]. The weight of each edge represents the confidence score of each putative interaction, as defined in ref[29]. These confidence scores range from 0.38 to 1. For AP, the input 'similarities' were again defined twice for every pair of interacting proteins i, j as S(i, j) = S(j, i) = c, where c is the confidence assigned to the interaction.

### Performance assessment

The ability of each clustering algorithm to recapitulate the known complexes from the weighted or unweighted interaction graph was measured using the Geometric Accuracy (*Acc*) and Geometric Separation (*Sep*), which are derived from a Confusion Table T, where each entry T_i, j _gives the number of proteins in common between complex i and cluster j[15]:





The *Acc *indicates the tradeoff between the Sensitivity and the Positive Predictive Value (PPV), and is calculated by taking the geometric mean of these two quantities. Sensitivity is defined as the weighted average complex-wise sensitivities, S_i_, and cluster-wise positive predictive values P_j_. S_i _measures the best overlap of complex i with the predicted clusters, and Pj measures the best overlap of cluster j with the gold standard complexes, relative to the number of components in cluster j that are contained in the original set of complexes. The *Acc *alone may not give an accurate evaluation of a clustering – for example, if the clustering consists of very large and very small clusters[15]. In this case both the complex-wise Sensitivity and cluster-wise PPV will be high.

A second measure, the *Sep*, is therefore calculated to measure the one-to-one correspondence between predicted clusters and complexes. It is defined as the geometric mean of the average complex-wise and average cluster-wise separation, which are each derived from confusion tables modified, respectively, to indicate the fraction of overlap of each complex with every cluster, or each cluster with every complex. Unlike Brohée et al, all calculations done here consider only those components that exist in both datasets.

### Graph Properties

The mass and area fractions were computed for each unweighted graph using the clminfo tool provided with the MCL implementation. The mass fraction measures the total weight of all edges (interactions) that occur between proteins within the same cluster, relative to the total weight of all edges. For an interaction network of edges, *E*, and the subset of these edges contained entirely within clusters E* ⊆ *E*, the mass fraction is given by:



where *w*(*e*) denotes the weight of edge e.

The area fraction is calculated by translating a clustering into an interaction graph by considering each cluster as a clique, and then dividing the number of clique edges by the number of edges within a full clique. For a graph with *N *nodes and a clustering of the graph containing *C *clusters, where the number of components in the *i*^*th *^cluster is given by *n*_*i*_:



### Parameter Optimization

Each clustering was performed with parameters that maximized the Geometric Accuracy and Separation. For MCL this involved sampling Inflation parameter values of 1.5 – 4 in steps of 0.1. For the AP algorithm we sampled the Preference parameters from 0.1–1 in steps of 0.05. The damping factor was set to 0.99, the maximum number of iterations to 15,000, and the number of iterations required for convergence to 1500. For AP, all proteins were assigned the same preference.

## Authors' contributions

JV participated in the design of the study and performed the analysis. SW assisted in the study design, analysis, and manuscript preparation. All authors read and approved the final manuscript.

## Supplementary Material

Additional File 1 **Curated Complexes.** Curated complexes[28] used to generate the unweighted networks, and taken as the reference set for computing the Geometric Accuracy (*Acc*) and Separation (*Sep*) values (see main text for detail). Click here for file

Additional File 2 **Clustering Results, Unweighted Network.** AP and MCL results for all parameters tested, at all noise levels for the unweighted network. Columns descriptions are listed in the 'col_descriptions' worksheet tab of this spreadsheet. See also reference [15] and main text for descriptions of *PPV, Sensitivity, Acc, SepCl, SepCo*, and *Sep*. Click here for file

Additional File 3 **MCL Human PPI Size Distribution.** Size distributions of clusters in partitions computed by MCL on a human PPI network (15 982 interactions and 5850 proteins) extracted from version 2.0.50 of the Biogrid database[33]. AP did not converge for this network, precluding any comparison. Click here for file

Additional File 4 **Supplementary Figures.** Supplementary figures referred to in the main text. Click here for file

Additional File 5 **Clustering Results, Weighted Network.** AP and MCL results for all parameters tested, at all noise levels for the weighted network. Column descriptions are given in the 'col_descriptions' worksheet tab of this spreadsheet. See also reference [15] and main text for descriptions of *PPV, Sensitivity, Acc, SepCl, SepCo*, and *Sep*. Click here for file
