# Supplementary material for: Markov clustering versus affinity propagation for the partitioning of protein interaction graphs
Source: BMC Bioinformatics. 2009 Mar 30;10:99. doi: 10.1186/1471-2105-10-99 (PMC2682798; doi:10.1186/1471-2105-10-99)
Supplement: Additional File 4 — Supplementary Figures. Supplementary figures referred to in the main text. [file 1471-2105-10-99-S4.doc]

**Markov Clustering versus Affinity Propagation for the**

**Partitioning of Protein Interaction Graphs**

**Supplementary Figures**

James Vlasblom^1,2^ and Shoshana J. Wodak*^1,2,3^

# ^1^ *Molecular Structure and Function Program, Hospital for Sick Children, 555 University Avenue, Toronto ON, Canada M5G 1X8*

# *^2^ Department of Biochemistry University of Toronto, 1 Kings College Circle, Toronto ON, Canada M5S 1A8*

# *^3^ Department of Molecular Genetics, University of Toronto, 1 Kings College Circle, Toronto ON,*

# *Canada M5S 1A8*

**Figure Captions**

**Figure S1:** Geometric accuracy (*Acc*) and separation (*Sep*) as AP preference (a) and MCL inflation (b) are varied for the weighted network with 40% of edges randomly shuffled. For AP, only points that converged are plotted.

**Figure S2:** Geometric Accuracy (*Acc*) for each clustering algorithm as measured against a subset of the gold standard complexes - consisting of all complexes of a certain size as indicated on X axis – for the weighted network with (a) no noise, and (b) 40% of edges shuffled.

**Figure S3:** Complexes differentially identified by AP and MCL.

(a) FBP degradation and (b) RNA Polymerase III complexes. Edges indicate associations between proteins predicted by Collins et al. Green edges indicate confidence scores greater than 0.5, and red edges indicate confidence scores in the range 0.38 – 0.5. Each complex was entirely recovered by the MCL algorithm, but not by AP. Red nodes indicate proteins assigned to the same cluster in both algorithms, green nodes indicate proteins assigned to a different cluster by AP, and white nodes indicate proteins discarded as singletons by AP.

**(a) (b)**

**
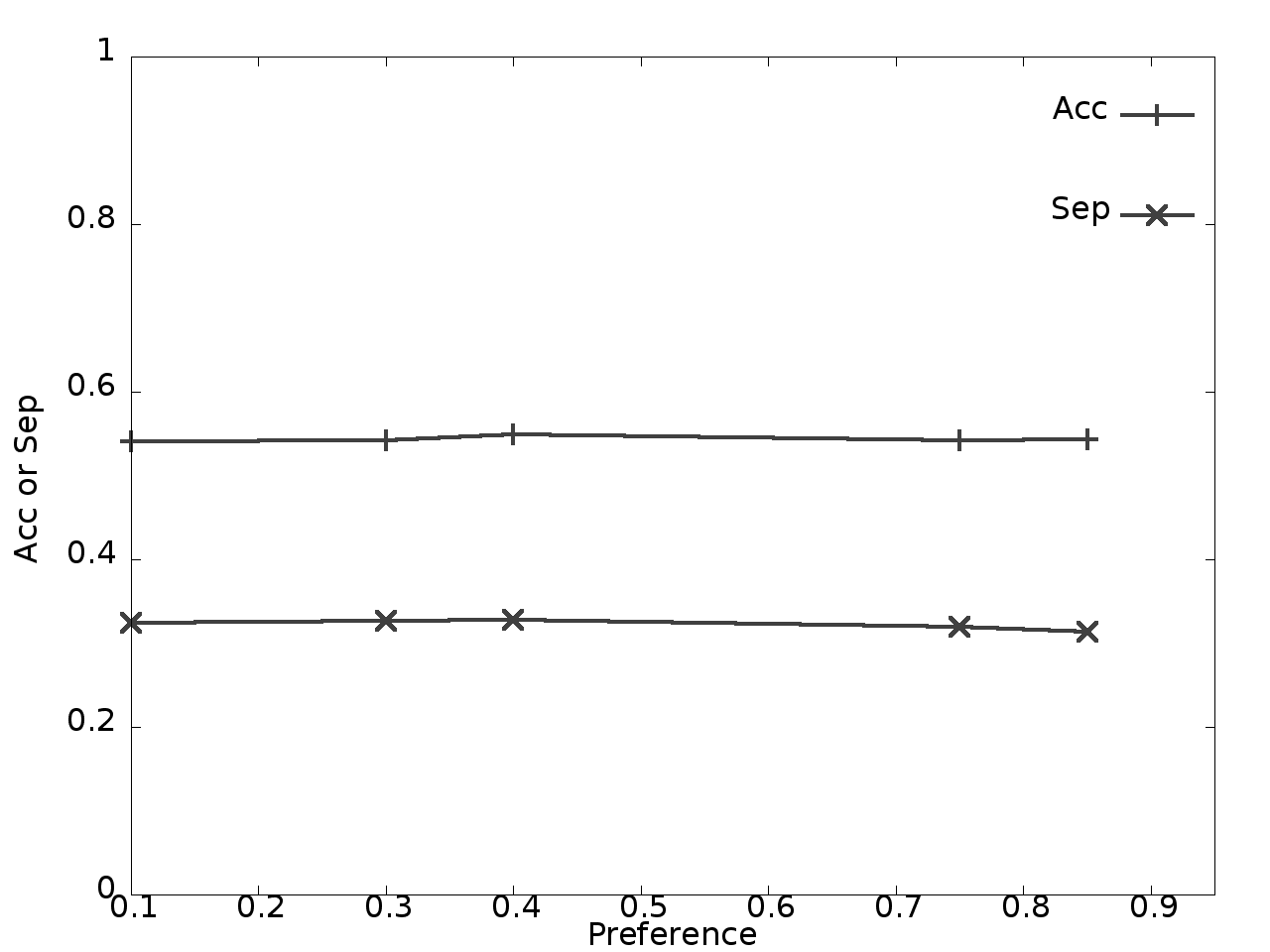

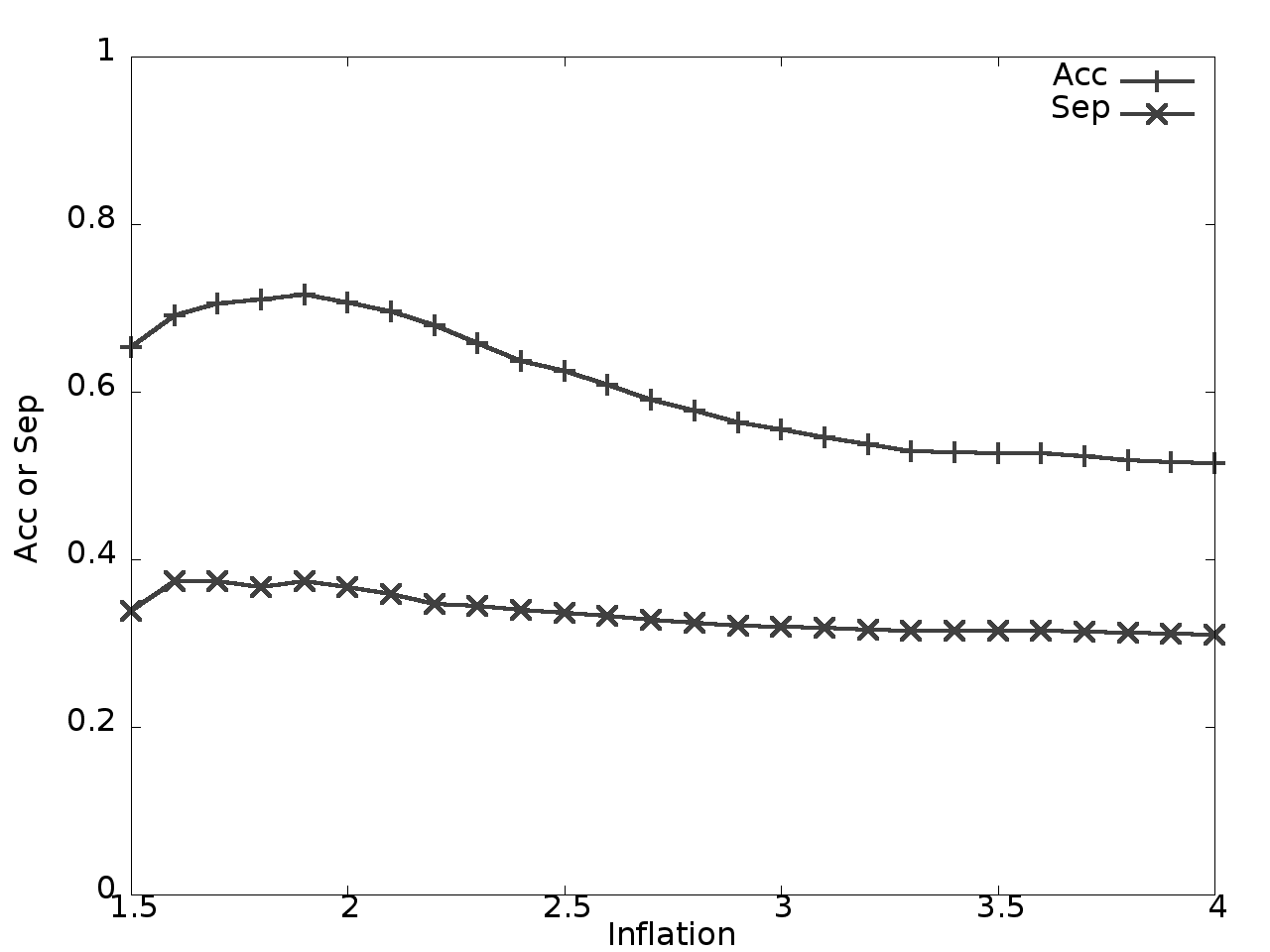
**

**Figure S1**

**(a) (b)**


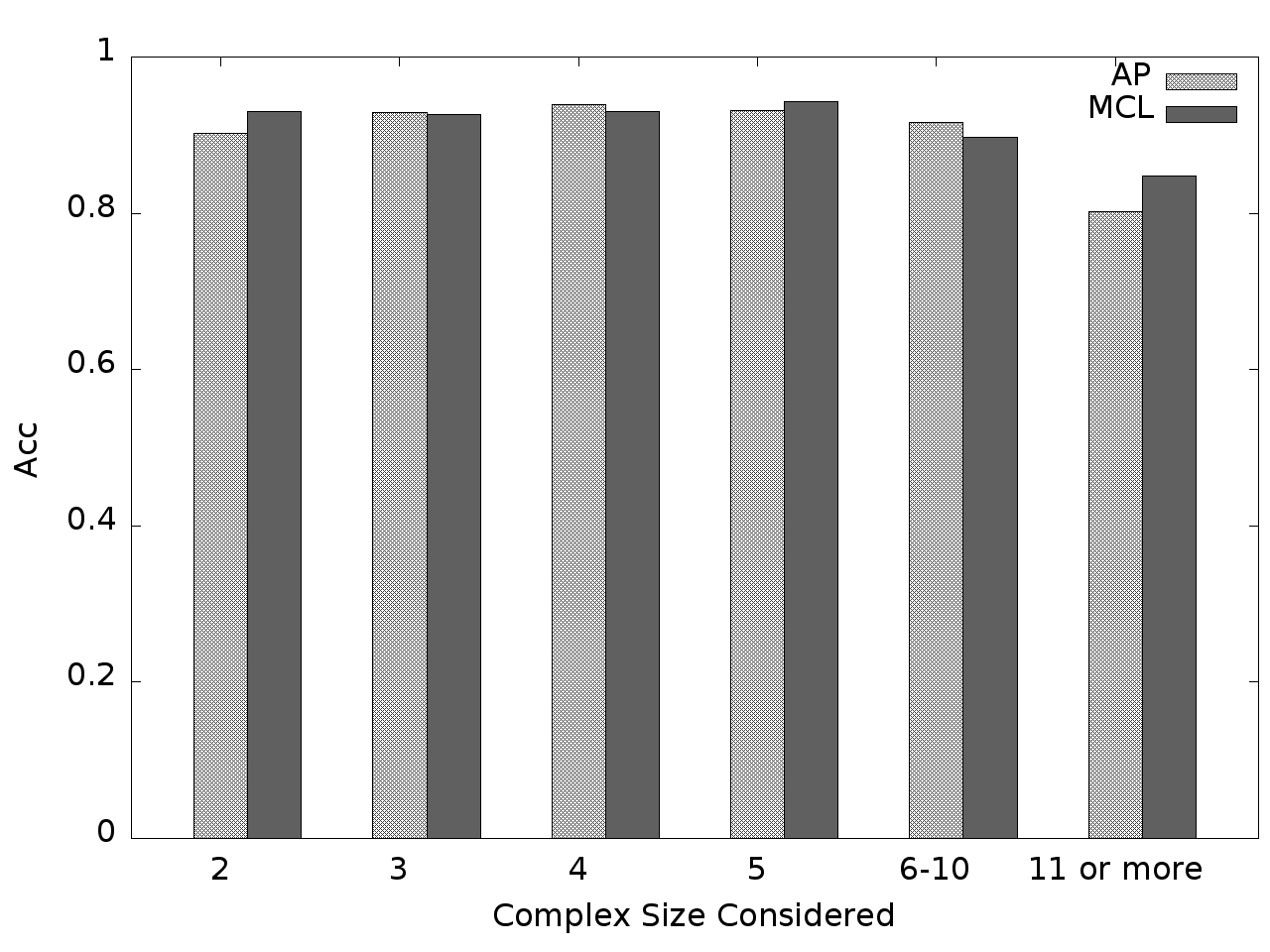

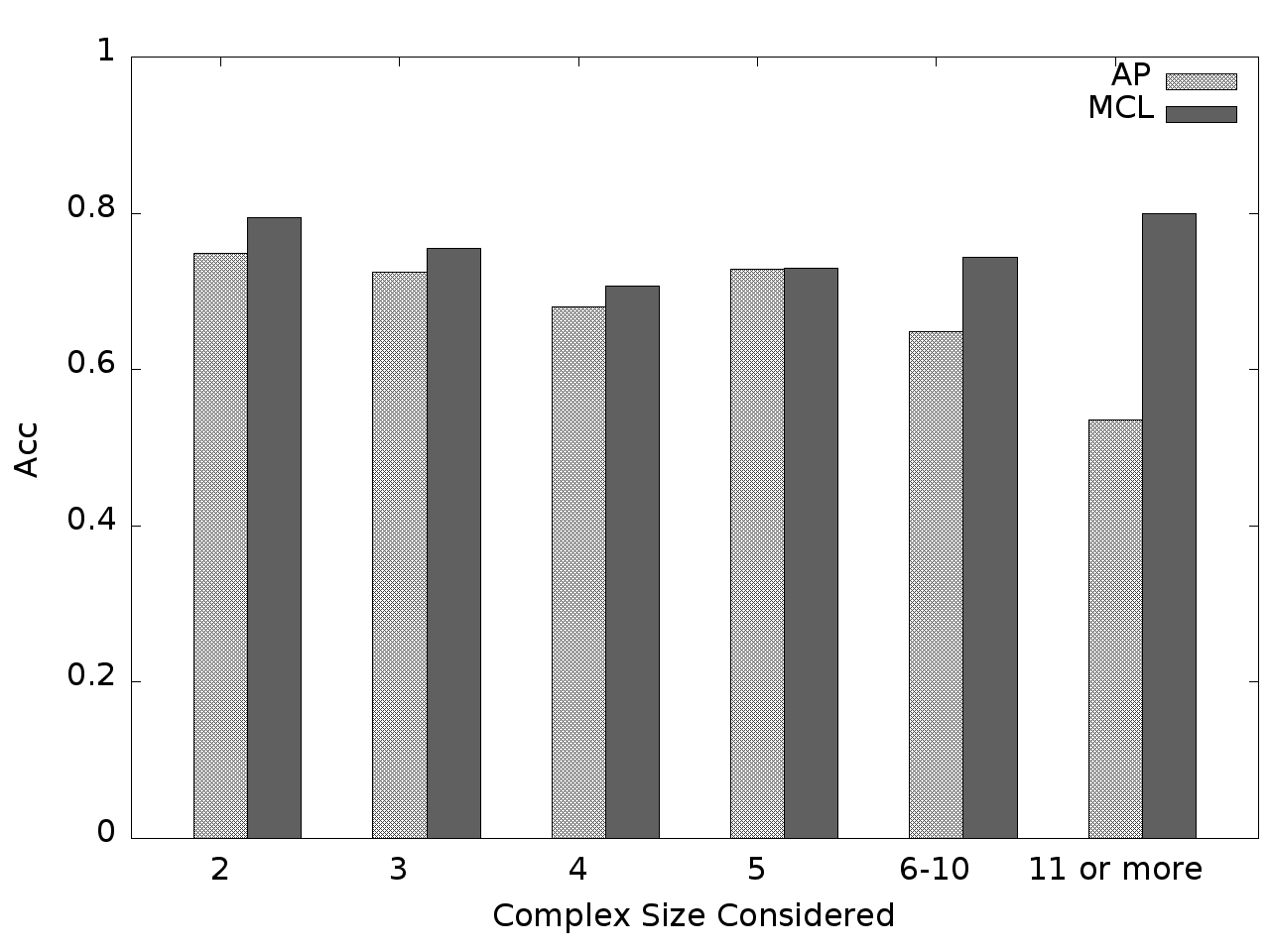


**Figure S2**

**a)** **b)**


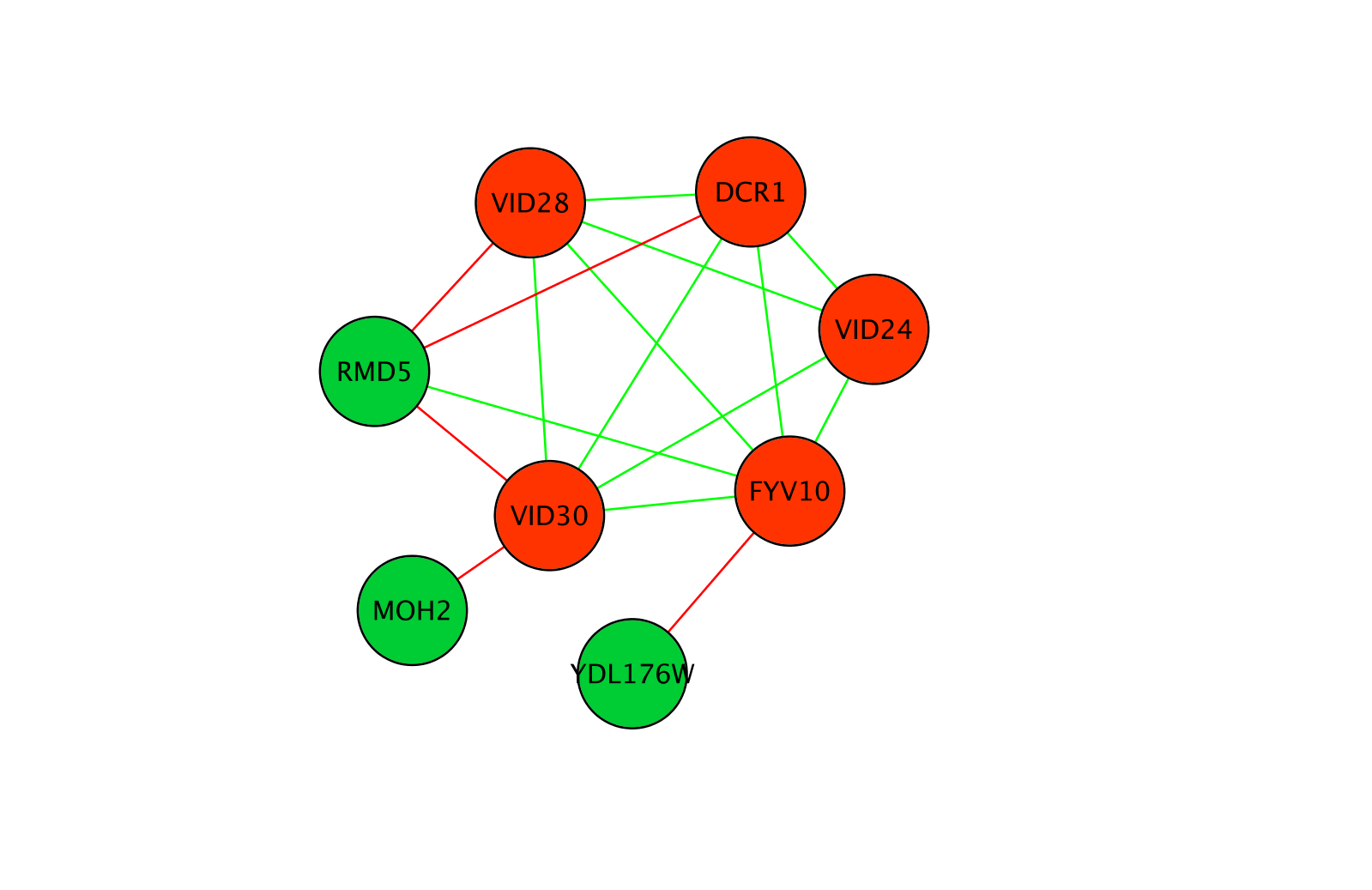

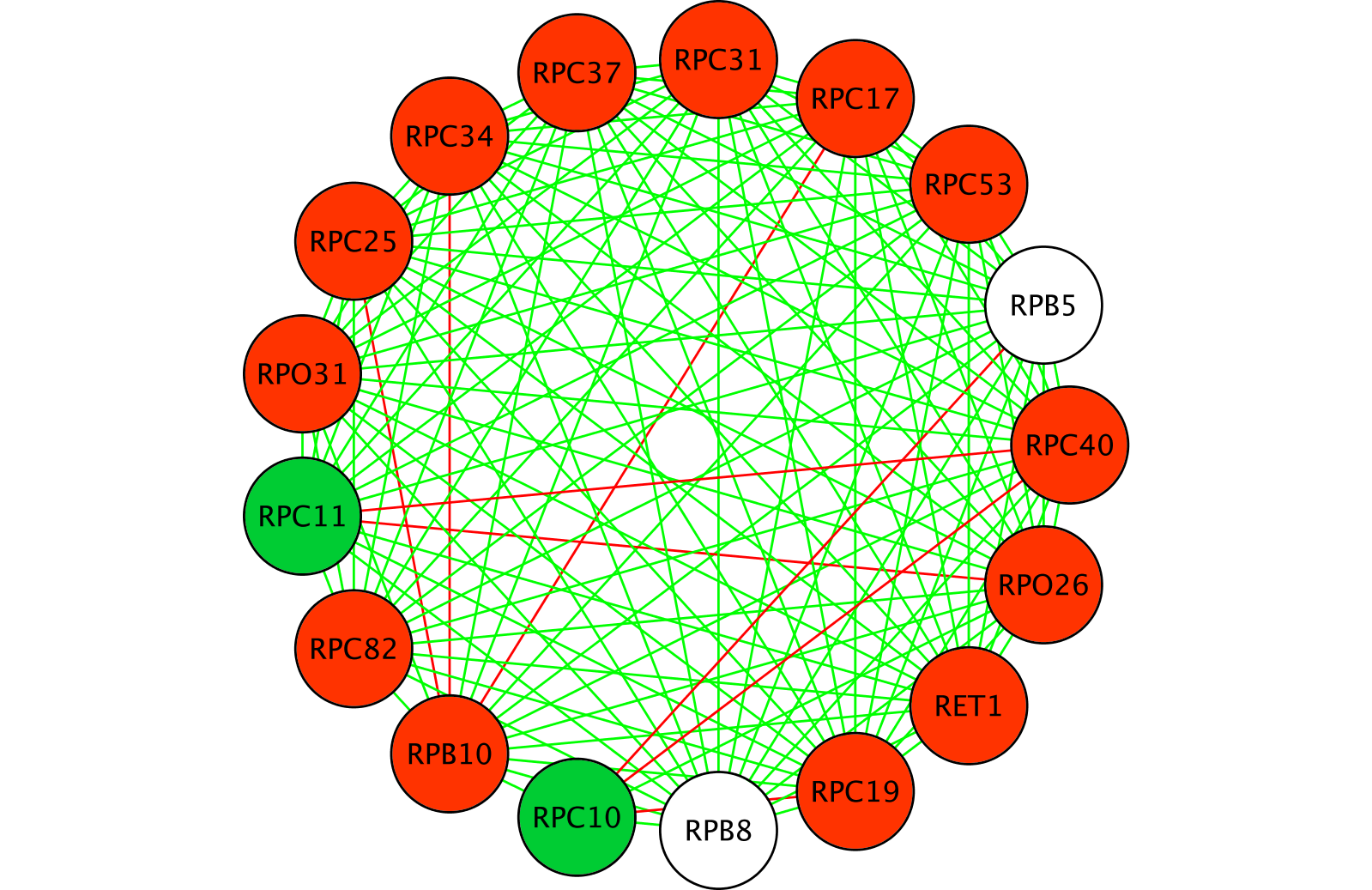


**Figure S3**
